# Independent risk factors and long-term outcomes for acute kidney injury in pediatric patients undergoing hematopoietic stem cell transplantation: a retrospective cohort study

**DOI:** 10.1186/s12882-020-02045-8

**Published:** 2020-08-27

**Authors:** Daishi Hirano, Daisuke Kakegawa, Saori Miwa, Chisato Umeda, Yoichi Takemasa, Ai Tokunaga, Yuhei Kawakami, Akira Ito

**Affiliations:** 1 Department of Pediatrics, The Jikei University School of Medicine, 3-25-8 Nishi-Shimbashi, Minato-ku, Tokyo, 105-0003 Japan; 2 Division of Nephrology, Saitama Children’s Medical Center, Saitama, Japan

**Keywords:** Acute kidney injury, Matched unrelated donor, pRIFLE, Child, Risk factor

## Abstract

**Background:**

Acute kidney injury (AKI) remains a frequent complication in children undergoing hematopoietic stem cell transplantation (HSCT) and an independent risk factor of the patient’s survival and a prognostic factor of progression to chronic kidney disease (CKD). However, the causes of these complications are diverse, usually overlapping, and less well understood.

**Methods:**

This retrospective analysis was performed in 43 patients (28 boys, 15 girls; median age, 5.5 years) undergoing HSCT between April 2006 and March 2019. The main outcome was the development of AKI defined according to the Pediatric Risk, Injury, Failure, Loss, End-stage Renal Disease (pRIFLE) criteria as ≥ 25% decrease in estimated creatinine clearance. The secondary outcome was the development of CKD after a 2-year follow-up.

**Results:**

AKI developed in 21 patients (49%) within 100 days after HSCT. After adjusting for possible confounders, posttransplant AKI was associated with matched unrelated donor (MUD) (HR, 6.26; *P* = 0.042), but not total body irradiation (TBI). Of 37 patients who were able to follow-up for 2 years, 7 patients died, but none had reached CKD during the 2 years after transplantation.

**Conclusions:**

Posttransplant AKI was strongly associated with HSCT from MUD. Although the incidence of AKI was high in our cohort, that of posttransplant CKD was lower than reported previously in adults. TBI dose reduced, GVHD minimized, and infection prevented are required to avoid late renal dysfunction after HSCT in children since their combinations may contribute to the occurrence of AKI.

## Background

The risks of chronic kidney disease (CKD), as well as short- and long-term mortality rates, are increased in patients who develop acute kidney injury (AKI) following hematopoietic stem cell transplantation (HSCT). AKI continues to show adverse health effects after hospital discharge, and the risks of CKD and long-term mortality were reported to be greater in patients with than without AKI [1]. AKI was reported to occur in 20 to 84% of pediatric patients undergoing HSCT [2–4]. AKI after HSCT has multifactorial etiologies with pre-renal, renal, and postrenal mechanisms, including conditioning chemotherapy, total body irradiation (TBI), nephrotoxic medications, sepsis, sinusoidal obstruction syndrome (SOS), thrombotic microangiopathy (TMA), and graft-versus-host disease (GVHD) [5–7]. With advances in supportive care, the prevalence of post-HSCT AKI had decreased over the past several years [8–12]. However, kidney injury remains a significant complication of HSCT, negatively affecting patients’ quality of life (QOL) and both early and long-term mortality rates. Therefore, to improve transplant outcomes, it is important to determine risk factors, understand the causes, and develop methods for the early diagnosis and treatment of kidney injury.

It was difficult to determine the epidemiology of pediatric HSCT-related AKI as previous studies used various definitions of AKI. Therefore, it is necessary to develop a consistent, uniform definition to gain insight into the epidemiology of pediatric AKI and to facilitate comparisons between different studies, which would lead to improvements in prognosis. The Pediatric Risk, Injury, Failure, Loss, End-stage Renal Disease (pRIFLE) criteria were developed in 2007 to address this issue [13]. Accurate determination of the incidence, risk factors, and prognosis of pediatric HSCT-related AKI will require the accumulation of studies based on these standardized criteria. Therefore, the present study was performed to examine the incidence of AKI after HSCT defined according to the pRIFLE criteria, to identify independent risk factors for the development of AKI, and to examine the association between AKI and progression to CKD to identify factors that contribute to AKI.

## Materials and methods

### Study population

This study was performed using data for all children undergoing HSCT between April 2006 and March 2019 at Jikei University School of Medicine, Japan, including information on age at disease onset, sex, comorbidities, age at transplantation, transplant characteristics (e.g., type of donor, graft source, HLA matching, method of GVHD prophylaxis), indications for HSCT, conditioning regimen, acute GVHD, sinusoidal obstruction syndrome (SOS), transplant-associated thrombotic microangiopathy (TMA), tumor lysis syndrome (TLS), nephrotoxic medication exposure, renal replacement therapy (RRT), AKI, and survival outcome. The exclusion criteria were age > 21 years old, history of prior dialysis, and missing pre- or post-HSCT height, body weight, and serum creatinine (sCr) values. Only the first HSCT was counted for patients that underwent more than one transplant during the study period.

### Outcome variables

The cumulative incidence and severity of AKI within 100 days after HSCT were taken as the primary endpoints in this study. In accordance with the pRIFLE criteria, AKI was defined as a decrease in the estimated glomerular filtration rate (eGFR) ≥ 25% [13]. Uemura’s equation was used to calculate sCr-based eGFR [14]. The baseline sCr was defined as the median of three extracted sCr levels measured during the four weeks before the conditioning regimen. As all urine output data were not available, pRIFLE class was determined based on the worst sCr level. To evaluate the effects of AKI severity on outcome, the maximum stage of AKI was defined as the highest stage observed within the first 100 days after HSCT. The secondary outcome was the development of CKD after a 2-year follow-up. According to the National Kidney Foundation-Kidney Disease Outcomes Quality Initiative guideline, CKD was defined as an eGFR < 90 mL/min/1.73m^2^ being persistent more than 3 months post-transplant [15]. Acute GVHD was graded according to the standard criteria [16]. The diagnosis and severity of SOS were defined according to the modified Seattle criteria [17]. Both acute GVHD and SOS were considered valid only if they developed before AKI and were included in our analysis to evaluate their effects on AKI.

### Statistical analysis

Patient and disease characteristics were summarized using descriptive statistics. Categorical variables were reported as frequencies and percentages and were compared using the chi-square test or Fisher’s exact test as appropriate. Continuous variables were reported as the median (interquartile range; IQR) and were compared using the Mann–Whitney *U* test. Survival data were analyzed using the Kaplan–Meier method and were compared across groups using the log-rank test [18, 19]. Multivariable analysis using the Cox proportional hazards model was performed to identify independent risk factors for the occurrence of AKI, and to adjust for variables such as sex, age at HSCT, donor source, TBI, and GVHD, predictors of AKI identified in previous studies including statistically significant variables on univariate analysis. In the Cox regression models, follow-up time was counted from the date of HSCT until the date on which the outcome was reached or the date of the last examination. All statistical analyses were performed using STATA 14.2 (StataCorp, College Station, TX). In all analyses, *P* <  0.05 was taken to indicate statistical significance.

## Results

### Baseline characteristics of the study population and prevalence of AKI

**Fig. 1:**
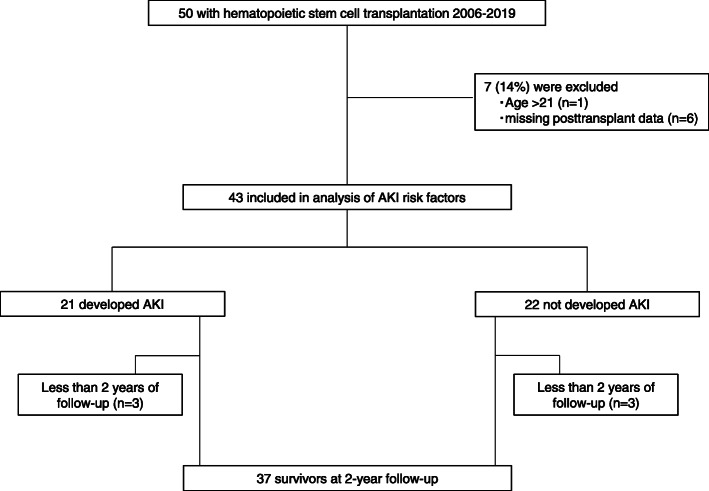
Study population. A total of 50 children underwent HSCT during the study period. Of these, 43 children fulfilled the inclusion criteria. Data regarding 2-year outcome were available for 37 patients

A total of 50 children underwent HSCT during the study period at our institute, and 43 of these children fulfilled the inclusion criteria. Data regarding 2-year outcomes were available for 37 of the 43 total patients (Fig. 1). Table 1 summarize the patient characteristics. Forty-three of 50 children undergoing HSCT at our institute during the study period met the inclusion criteria for this study. The median age of the study population at the time of HSCT was 5.5 years (IQR, 4.1–10.8), and 65% were boys. Stem cells for transplantation were obtained from bone marrow (*n* = 19, 44%), peripheral blood (*n* = 21, 49%), or cord blood (*n* = 3, 7%), and were from autologous (*n* = 20, 46%), matched related donor (MRD), (*n* = 5, 12%), and matched unrelated donor (MUD) (*n* = 18, 42%) as the source. Twenty-one patients (49%) developed AKI within the first 100 days after HSCT, and the median time between transplantation and AKI was 38.0 days (IQR, 26.0–63.0). Using the pRIFLE criteria, 12 (28%) patients were at risk of renal dysfunction, 5 (12%) patients had injury to the kidney, and 4 (9%) had failure of kidney function. Two cases (5%) in the total cohort underwent postoperative dialysis.

**Table 1:** Patient characteristics

	Whole cohort(***n*** = 43)	AKI(***n*** = 21)	Non-AKI(***n*** = 22)	***P***-value
**Age at HSCT, years, median (IQR)**	5.5 (4.1–10.8)	5.5 (4.2–14.4)	5.2 (4.1–7.8)	0.37
**Sex, Male,** ***n*** **(%)**	28 (65)	14 (67)	14 (64)	0.84
**Indication for HSCT**				
Malignant	34 (79)	18 (86)	16 (73)	0.46
**Conditioning regimen,** ***n*** **(%)**				
Calcineurin inhibitor	4 (9)	2 (10)	2 (9)	1.00
TBI use	22 (51)	15 (71)	7 (32)	< 0.01
**Stem cell source,** ***n*** **(%)**				
Bone marrow	19 (44)	9 (43)	10 (45)	0.86
Peripheral blood	21 (49)	9 (43)	12 (55)	0.44
Umbilical cord	3 (7)	3 (14)	0 (0)	0.11
**Donor source,** ***n*** **(%)**				
Autologous	20 (46)	7 (33)	13 (59)	0.091
MRD	5 (12)	1 (5)	4 (18)	0.35
MUD	18 (42)	13 (62)	5 (23)	< 0.01
**Acute GVHD,** ***n*** **(%)**	20 (47)	12 (67)	8 (36)	0.17
I	8 (19)	4 (18)	4 (18)	1.00
II – IV	12 (28)	8 (38)	4 (19)	0.19

HSCT, hematopoietic stem cell transplantation; TBI, total body irradiation; MRD, matched related donor; MUD, matched unrelated donor; GVHD, graft-versus-host disease

### Factors associated with the development of AKI

**Fig. 2:**
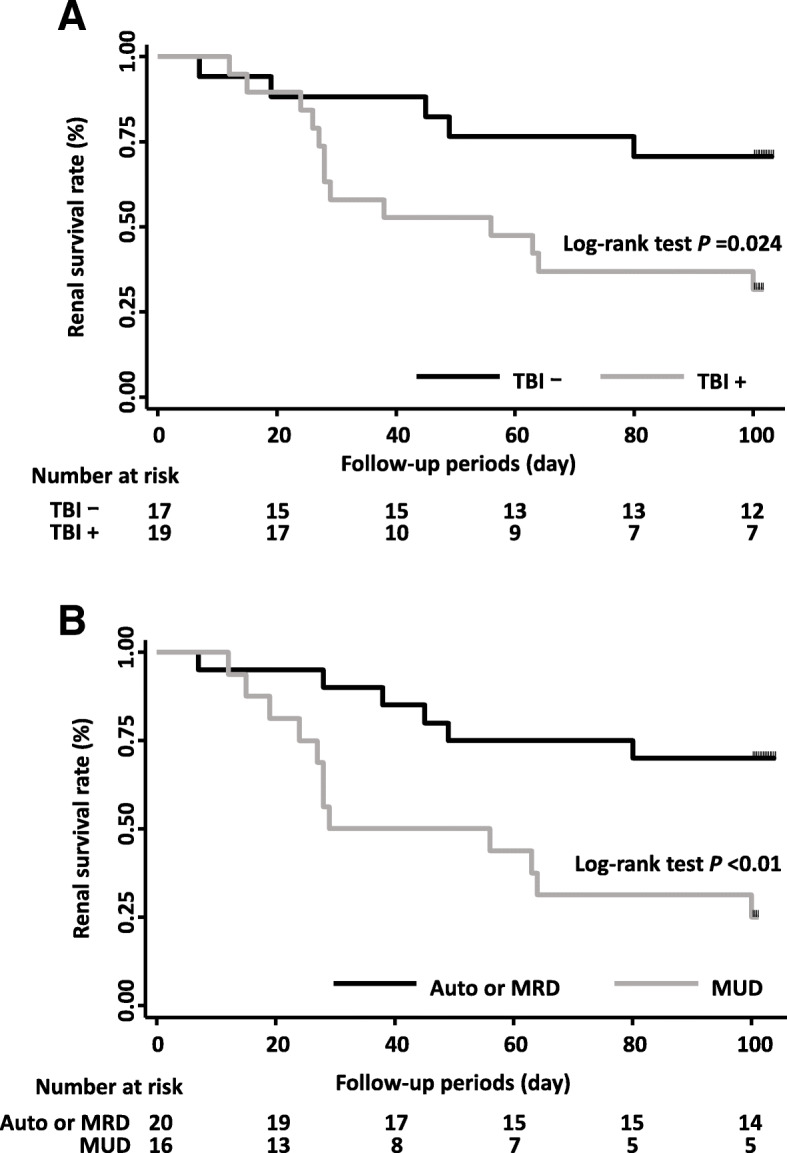
AKI development probability graph according to the survival analysis and log-rank test. AKI development probabilities were higher in patients with than in those without TBI or MUD (Fig. 2a, Fig. 2b, respectively)

On univariate analysis, the development of AKI after HSCT was significantly associated with TBI and MUD (*P* <  0.01, *P* <  0.01, respectively). There were no significant differences in age at the time of transplant, sex, indication for HSCT, use of calcineurin inhibitors, or presence of aGVHD between patients with and without AKI. On Kaplan–Meier survival analysis, the cumulative incidence of AKI was higher in patients with TBI or MUD than in those without TBI or MUD (*P* = 0.012, *P* <  0.01, respectively) (Fig. 2a, b). After adjustment for possible confounding factors, posttransplant AKI was associated with MUD (*P* = 0.042), but not with TBI use (Table 2).

**Table 2:** Cox hazard regression analysis of possible risk factors for posttransplant AKI

	95% confidence interval (CI)			
	Hazard ratio	Lower	Upper	***P***-value
**Sex (male)**	1.11	0.43	2.82	0.83
**Age (> 10 years)**	0.96	0.38	2.45	0.93
**Donor source (MUD)**	6.26	1.07	36.76	0.042
**TBI use**	2.18	0.63	7.60	0.22
**Acute GVHD**	0.25	0.05	1.24	0.09

MUD, matched unrelated donor; TBI, total body irradiation**;** GVHD, graft-versus-host disease

### Two-year outcomes of patients with Posttransplant AKI

Data regarding 2-year outcomes were available for 37 of the 43 total patients. Seven patients (19%) in the total study population died during the 2-year follow-up period due to progression of primary disease (*n* = 5), infection (*n* = 1), and end-stage renal disease (*n* = 1). None of the children who survived had chronic renal insufficiency 2 years after HSCT. The groups with and without AKI showed 2-year overall survival (OS) rates of 81 and 86%, respectively, which were not significantly different (*P* = 0.70).

## Discussion

The present observational study was performed to investigate the incidence of AKI defined according to the pRIFLE criteria following HSCT in pediatric patients. We also identified independent risk factors for the development of AKI and evaluated the associated risk of CKD in this clinical setting.

AKI occurred within 100 days of transplantation at a rate of 49% in this study population. Thus, AKI appears to be common in pediatric HSCT recipients. Moreover, we also found that the occurrence of various degrees of AKI spread after HSCT. The reported incidence rate of AKI after HSCT in pediatric patients shows a wide range of 21–84% due primarily to the use of different definitions of AKI and also the large degree of heterogeneity in patient characteristics [3, 9, 20–22]. The pRIFLE criteria were used in the present study, which allowed a fuller understanding of the epidemiology of pediatric AKI. As the pRIFLE criteria can detect renal dysfunction in the early stages, which detection of AKI at a high rate of 49% in the present study, similar to previous reports [3, 23].

Several risk factors for AKI in patients undergoing HSCT have been reported. The descriptions of transplant characteristics, such as donor, race, TBI, nephrotoxic agents, and post-transplant adverse events, such as SOS and VOD, were inconsistently described as risk factors for the development of AKI in pediatric patients [3, 11, 20–25]. After adjusting for possible confounding variables by logistic regression analysis, HSCT from unrelated donors was shown to be an independent risk factor for posttransplant AKI in the present study, whereas sex, age, TBI, and acute GVHD were not related to the incidence of AKI. Unrelated donor HSCT was reported previously to be related to significant increases in the risk of infections, severe aGVHD, and organ toxicities [26–31]. Although these complications may not represent independent risk factors for AKI, their combinations may contribute to the occurrence of AKI. In addition, the nephrotoxic agents used in the treatment of these complications can also lead to AKI.

In contrast to previous studies in adult patients, chronic renal insufficiency 2 years after HSCT was not correlated with AKI in our cohort. None of the pediatric patients included in the present study had chronic renal insufficiency 2 years after HSCT, despite the high frequency of AKI. The cumulative incidence rate of CKD in adult patients after HSCT was reported to vary from 13 to 66% [32–35]. Similar to our findings, other studies reported CKD prevalence rates of 8–11% per year in pediatric patients [3, 21, 22]. Risk factors for posttransplant CKD include previous AKI, acute and chronic GVHD, age ≥ 45 years at the time of transplantation, baseline eGFR < 90 ml/min/1.73 m^2^, hypertension, and exposure to high-dose TBI [22, 36–39]. Although TBI has been suggested to induce artery and capillary sclerosis, interstitial fibrosis, and to play a major role in renal damage [40–44], Kal et al. [45] reported that late renal dysfunction can be avoided by reducing the strength of the TBI regimen to < 16 Gy and by using appropriate kidney shielding. Since the development of a reduced-intensity TBI regimen (< 10 Gy), TBI was not an etiological pathogenetic factor in the present study in contrast to previous reports showing dose-dependent toxicity, even in pediatric patients [36, 46]. In addition, the favorable outcome in the present study may also have been related to recent progress in supportive care, management of GVHD, and HLA typing techniques.

This study had several limitations, the first of which is related to its retrospective nature in pediatric patients undergoing HSCT at a single center, which may have resulted in a degree of selection bias. The single-center cohort design also limited the external validity of our findings. Besides, since we were not able to obtain data about previously well-known risk factors such as prior AKI, severe infection, nephrotoxic antibiotics or anti-fungal agents, and contrast dye for computed tomography, we could not conduct more in-depth analyses for identifying risk factors predicting AKI. Second, as previously shown by Kaddourah A et al., decreased urine output without a significant change in sCr is a massive determinant of AKI in critically ill children [47]. Therefore, AKI was possibly underestimated in this study, since we could not obtain the data of urine output. In addition, the accuracy and validity of this work are further limited by the use of the “worst sCr level” because such level could have been abnormally related to prior interventions that were part of the delivered care that resulted in the need for HSCT. Third, the follow-up period of 2 years in this study was too short to allow documentation of all outcomes. However, in most studies reported to date, 70% of cases of CKD occurred within 2 years (especially within 6 months), and the incidence of CKD > 2 years after transplantation is relatively rare [48, 49]. Fourth, low statistical power and model overfitting may have been existent due to the limited sample size.

## Conclusion

The results presented here indicated that the occurrence of posttransplant AKI was strongly associated with HSCT from unrelated donors. Despite the high rate of posttransplant AKI, the rate of posttransplant CKD was lower than in previous studies in adult patients. The dose of TBI should be reduced, and kidney shielding should be applied to avoid the occurrence of late renal dysfunction after HSCT.

## Data Availability

The datasets used and analyzed during the current study are available from the corresponding author.
